# Emotion Recognition from Realistic Dynamic Emotional Expressions Cohere with Established Emotion Recognition Tests: A Proof-of-Concept Validation of the Emotional Accuracy Test

**DOI:** 10.3390/jintelligence9020025

**Published:** 2021-05-07

**Authors:** Jacob Israelashvili, Lisanne S. Pauw, Disa A. Sauter, Agneta H. Fischer

**Affiliations:** 1 Psychology Department, The Hebrew University of Jerusalem, Jerusalem 9190501, Israel; 2 Department of Psychology, University of Münster, 48149 Münster, Germany; lpauw@uni-muenster.de; 3 Faculty of Social and Behavioral Sciences, Department of Psychology, University of Amsterdam, 1001 NK Amsterdam, The Netherlands; D.A.Sauter@uva.nl (D.A.S.); a.h.fischer@uva.nl (A.H.F.)

**Keywords:** emotion recognition, emotional accuracy, empathy, individual differences

## Abstract

Individual differences in understanding other people’s emotions have typically been studied with recognition tests using prototypical emotional expressions. These tests have been criticized for the use of posed, prototypical displays, raising the question of whether such tests tell us anything about the ability to understand spontaneous, non-prototypical emotional expressions. Here, we employ the Emotional Accuracy Test (EAT), which uses natural emotional expressions and defines the recognition as the match between the emotion ratings of a target and a perceiver. In two preregistered studies (N_total_ = 231), we compared the performance on the EAT with two well-established tests of emotion recognition ability: the Geneva Emotion Recognition Test (GERT) and the Reading the Mind in the Eyes Test (RMET). We found significant overlap (*r* > 0.20) between individuals’ performance in recognizing spontaneous emotions in naturalistic settings (EAT) and posed (or enacted) non-verbal measures of emotion recognition (GERT, RMET), even when controlling for individual differences in verbal IQ. On average, however, participants reported enjoying the EAT more than the other tasks. Thus, the current research provides a proof-of-concept validation of the EAT as a useful measure for testing the understanding of others’ emotions, a crucial feature of emotional intelligence. Further, our findings indicate that emotion recognition tests using prototypical expressions are valid proxies for measuring the understanding of others’ emotions in more realistic everyday contexts.

## Highlights

- A positive relation was found for the recognition of posed, enacted and spontaneous expressions.
- Individual differences were consistent across the three emotion recognition tests.
- Participants most enjoyed the test with real emotional stories (EAT).

## 1. Introduction

Scholars in different research traditions have argued that the ability to understand the emotions of other people is essential for successful interpersonal relationships (e.g., Elfenbein et al. 2007; Fischer and Manstead 2016; Hall et al. 2009; Salovey and Mayer 1990). Individuals who understand others’ emotions can respond to them effectively. Indeed, problems with understanding others’ emotions, a common feature of many psychopathologies, often coincide with problems in interpersonal relationships (Halberstadt et al. 2001; Hall et al. 2009; Hampson et al. 2006; Elfenbein et al. 2002, 2007). Due to the crucial role of understanding others’ emotions in social relationships, various tests have been developed to index individual differences in the ability to understand others’ emotions. This work has tended to use prototypical emotional facial expressions created in a lab context. Here, we take the first step towards validating a new measure that differs from existing tests in several ways: the Emotional Accuracy Test (EAT) assesses emotion recognition from spontaneous, multi-modal emotional expressions reflecting real-life emotional situations. We compare performance on the EAT with existing measures of emotion recognition, and also examine participants’ enjoyment of the different tests. 

### 1.1. Assessing Individual Differences in Emotion Recognition

Various emotion recognition tests have been developed to assess how well people recognize others’ emotions (see Israelashvili et al. 2019a; Schlegel et al. 2019). These tests commonly use stylized stimuli of brief static or dynamic posed emotional expressions. The expressions show stereotypical configurations of facial movements of the so-called basic emotions. Recently, tests using other nonverbal channels, such as bodily movements, or the voice, have been developed as well, often including a broader range of different emotions (e.g., Emotion Recognition Index; Scherer and Scherer 2011; Geneva Emotion Recognition Test; Schlegel et al. 2014). However, existing tests nevertheless make use of brief, posed emotional expressions as stimuli. 

Although the use of posed expressions allows researchers a high degree of experimental control, the use of posed expressions can inflate recognition accuracy rate relative to spontaneous expressions (e.g., Nelson and Russell 2013; but see Sauter and Fischer 2018). Moreover, concerns have been raised over whether perceivers can reliably recognize emotions from spontaneous expressions at all (Russell 1994), though some studies have shown good accuracy of spontaneous emotion expressions (e.g., Sauter and Fischer 2018; Wagner 1990). Posed stimuli have also been criticized for being artificial and, consequently, not representative of expressions that occur in real life (Barrett et al. 2019; Israelashvili et al. 2019b; Scherer et al. 2011). It is, however, unclear whether participants scoring highly on standardized emotion recognition tests are especially good at recognizing emotion prototypes, or whether they are also able to understand others’ emotions in everyday life. Previous research has compared recognition rates for the recognition of spontaneous vs. posed emotional expressions (e.g., Sauter and Fischer 2018), but this research did not include frequently used tests. Therefore, it is still unclear whether recognition tests using both posed and spontaneous stimuli derived from everyday emotional life experiences are based on a shared underlying ability. 

A second concern that has been raised about existing emotion recognition tasks is that verbal information is mostly absent (Hall and Schmid Mast 2007). This is remarkable, because humans often express their emotions verbally, for example, by scolding others, requesting help, or proclaiming their affection. In fact, we have a remarkably strong inclination to verbally share emotional events with others by telling others about our affective experiences (for a review, see Rimé 2009). In such narratives, the person sharing their experience typically explains what happened, what they thought, and how it made them feel and why. Such verbal narratives are often accompanied by non-verbal expressions. Thus, in daily life, observers typically have non-verbal and verbal information available when trying to understand others’ emotions, whereas, while the recognition of emotions from decontextualized expressions using only one modality can provide essential knowledge about the role of specific kinds of information for emotional communication, it may not capture how well people recognize emotions in more complex and multi-faceted daily life situations. 

To address these concerns, several recognition tasks have recently been developed that feature a combination of verbal and non-verbal emotional cues. The stimuli in these new recognition tasks are based around autobiographical emotional stories. For example, the *Empathic accuracy* paradigm^1^ (Zaki et al. 2008; Ta and Ickes 2017; Ong et al. 2019) assesses the perceiver’s sensitivity to changes in the affective valence of a target person sharing an emotional event. In that paradigm, participants judge the target’s feelings in terms of broad valence evaluations, namely degrees of positivity or negativity (see, e.g., Ickes et al. 1990; Rauers et al. 2013; Wilhelm and Perrez 2004) but are not asked to differentiate between emotions of the same valence. Building on this work, we recently developed a new measure, the *Emotional Accuracy Test* (EAT: see Israelashvili et al. 2020a)^2^. For this test, targets were videotaped sharing autobiographical emotional events from their own life. Afterward, they watched their own video and rated the intensity of 10 different emotions they experienced when telling the story they shared. Next, naive participants are asked to watch the videos and to rate the targets’ emotions on the same list and with the same scales as the targets. The similarity between the targets’ emotion scores and participants’ emotion scores constitute emotional accuracy. The key characteristic of the Emotional Accuracy Test is thus that it takes the experienced emotions of the target—rather than the intended emotion underlying posed expressions—as the basis of accuracy. The test thus measures a perceiver’s ability to identify a target’s emotions based on multiple types of information (e.g., words, vocal cues, facial and bodily movements) embedded in stories about a genuine emotional event. Both the stimuli and the task arguably map onto daily life situations, such as when people share experiences through video communication. 

Yet, in order to examine whether different tests rely on a shared underlying ability, performance with different measures need to be compared within a sample. Emotion recognition ability has been argued to rely on some domain-general abilities (Connolly et al. 2020; Schlegel et al. 2012, 2017; Lewis et al. 2016), suggesting that the nature of the stimuli and tasks should not matter much. However, comparisons of performance across different types of emotion recognition tests using the same sample are rare. One relevant meta-analysis showed that *nonverbal* emotion recognition tests significantly positively correlated with one another (i.e., an average correlation of *r* = 0.29; Schlegel et al. 2017). In the current study, we examined whether the EAT, using rich autobiographical stimuli with verbal and nonverbal cues, taps the same underlying process as measured by tests using posed, nonverbal stimuli.

When comparing different types of tests, it is also important to consider participants’ experience of the test, because this may affect test results. The use of repetitive, posed expressions may lead to a lack of concentration because such judgments are not enjoyable to engage in. Reduced enjoyment can be problematic and have various negative implications for test results (DeRight and Jorgensen 2015). Based on this rationale, we hypothesized that a test using real autobiographical stories, such as the EAT, would be more enjoyable than using posed expressions.

### 1.2. The Current Research

The current research aims to test the convergent validity of the Emotional Accuracy Test (EAT; Israelashvili et al. 2020a). This test is based on *dynamic*, *naturalistic* videos of targets who share emotional stories from their own lives in a way that resembles real-life situations when people use video calls. In other words, the stimuli are not posed, and the emotion displays make use of both verbal content and non-verbal signals. 

In order to test convergent validity, we compared performance on the EAT with two measures that are commonly used to test emotion recognition: the Reading the Mind in the Eyes Test (RMET; Baron-Cohen et al. 2001) and the Geneva Emotion Recognition Test (GERT; Schlegel et al. 2014). See Table 1 for description of these emotion recognition tasks. The RMET consists of *static*, *posed* pictures with minimal emotional information (only eyes). Although the RMET was originally designed to measure the theory of mind (ToM), it correlates strongly with other emotion perception tests, leading recent studies to discuss the RMET as a measure of emotion recognition, and not only of the ToM (for more details, see Oakley et al. 2016; Wilhelm et al. 2014). The GERT consists of *dynamic*, *reenacted* stimuli with different nonverbal channels (face, body, and voice). Both the RMET and GERT cover a relatively broad range of emotions. The EAT differs from the RMET and GERT in three ways. First, the RMET and the GERT do not include verbal cues, whereas the EAT does. Second, the stimuli in the GERT and RMET are posed or enacted, while in the EAT, they are spontaneous. Third, the tests differ in response format: the RMET provides four and the GERT fourteen multiple-choice options. The EAT uses rating scales, one for each relevant emotion (ten in total). 

By comparing performance on the EAT with the other two measures in the same sample, we sought to conduct a robust test of whether emotion recognition tests using prototypical expressions are valid proxies for measuring understanding of others’ emotions in more realistic daily life contexts. Finally, because emotion recognition tasks rely on vocabulary (e.g., Olderbak et al. 2015; see also supplemental materials in Israelashvili et al. 2020b), we also measured individual differences in verbal IQ in order to test whether the potential relation between the three tests would hold even when individual differences in verbal IQ were statistically controlled.

**Hypothesis** **1** **(H1).** *We hypothesized that all three recognition tests would be significantly and positively correlated.*

**Hypothesis** **2** **(H2).** *We further predicted that participants would enjoy the EAT significantly more than the GERT and the RMET.*

Our hypotheses were tested in two studies across two independent samples. The studies, including hypotheses, exclusion criteria, and analysis plan, were preregistered (Study 1: https://aspredicted.org/blind.php?x=hu2w6g, accessed on 14 May 2020; Study 2: https://aspredicted.org/blind.php?x=kq67vw, accessed on 4 November 2020). As Study 2 was a replication that used exactly the same procedure and measures, we report the studies together.

## 2. Method

### 2.1. Participants

*Study 1.* Participants were 161 US citizens, who were high reputation workers (above 95% of previously approved tasks) recruited via Amazon Mechanical Turk (Mturk). Seventy participants were excluded from the analyses because they performed below chance level^4^ on one or more tests (recognition tasks or verbal IQ). Eighteen participants were removed because they failed to correctly answer questions measuring attentiveness to the survey instructions. The remaining sample consisted of 74 US citizens (M_age_ = 38, SD_age_ = 12; 46% female, 54% male). 

*Study 2.* Participants were 200 UK citizens, who were high reputation workers (above 95% of previously approved tasks) recruited via Prolific Academic. Following our preregistered criteria, we removed (a) seven participants because they performed below chance level on one or more recognition test; (b) two participants because they did not spend the minimal time required to perform the test seriously; (c) four participants because they reported technical problems with watching or listening to the videos (resulting from disabled JavaScript on their computers); (d) thirty participants because they failed to correctly answer questions measuring attentiveness to the instructions of the survey. The remaining sample consisted of 157 UK citizens (M_age_ = 36, SD_age_ = 11; 64% female, 36% male).

In both studies, all the participants were currently living in an English-speaking country; for 88% of participants, English was their native language (72/74 in Study 1 and 138/157 in Study 2). A sensitivity analysis conducted in G-power suggested that with the standard criterion of α = 0.05, the analysis of correlations had a power of 0.80 to detect a medium effect (*r* = 0.3) in Study 1 and a small to medium effect (*r* = 0.2) in Study 2. The Ethics Committee of the Faculty of Social and Behavioral Sciences of the University of Amsterdam approved the study (EC 2020-SP-12183), and we obtained informed consent from all participants. 

### 2.2. Measures 

Reading the Mind in the Eyes Test (RMET). The RMET comprises 36 black and white photos depicting the eye region of 36 white individuals (Baron-Cohen et al. 2001). Participants are asked to identify the emotional state of each target by choosing one out of four words that each represents an emotional state (e.g., serious, ashamed, alarmed, or bewildered). Response options differ across the stimuli. Responses are scored as correct (1) or incorrect (0); the RMET score is calculated by summing the correct answers. The performance was determined by calculating the percentage of correct responses. The average accurate recognition in Study 1 was 62% (SD = 19%; Cronbach’s α = 0.84) and 72% in Study 2 (SD = 13%; Cronbach’s α = 0.69).

Geneva Emotion Recognition Test (GERT). We used the short version of the Geneva Emotion Recognition Test (Schlegel et al. 2014). The test consists of 42 short video clips (duration 1–3 s), in which ten white professional actors (five male, five female) express 14 different emotions: joy, amusement, pride, pleasure, relief, interest, surprise, anger, fear, despair, irritation, anxiety, sadness, and disgust. In each video clip, the actor is visible from their upper torso upward (conveying facial and postural/gestural emotional cues) and pronounces a nonsense-sentence (a series of syllables without semantic meaning). After each clip, participants were asked to choose which one out of the 14 emotions best describes the emotion the actor intended to express. Responses were scored as correct (1) or incorrect (0). Similar to the RMET, the final GERT score was calculated as the percentage of accurate recognition scores. The average recognition level in Study 1 was 38% (SD = 15%; Cronbach’s α = 0.81) and in Study 2 48% (SD = 11%; Cronbach’s α = 0.60).

Emotional Accuracy Test (EAT). In the Emotional Accuracy Test (Israelashvili et al. 2020a), participants watched four video clips in random order. Each video is between two and three minutes long and consists of an English-speaking woman in her early 20s who describes a genuine emotional autobiographical experience. The targets were asked to talk about an emotional experience from their own life that they felt comfortable sharing. The topics of the four videos were: (1) a parent being ill; (2) a divorced father in a new relationship; (3) emotional distance from family; and (4) problems with an internship (identical to the those used in Israelashvili et al. 2020a; Studies 3 and 4; researchers can contact the corresponding author if they want to use these stimuli for research). Each target showed sufficient variability in the reported intensity of her emotions (the variance between the emotions ranged from 2 to 6 intensity points for each target). Participants were asked to watch the videos and to rate the intensity with which the target had experienced each of ten emotions (anger, rage, disappointment, fear, sadness, worry, confusion, surprise, embarrassment, and guilt). Answers were given on a 7-point Likert scale, ranging from 0 = *not at all*; to 6 = *very much*. The targets’ own ratings were obtained by asking them to watch their videos (immediately after sharing the event) and to provide ratings of the emotions they felt in the video (“*How would you describe the emotions you have been feeling in the video? For each feeling listed below, indicate whether you were feeling it by moving the slider. If you think a certain label does not apply, you can leave it on the “not at all” position.*”). The emotion ratings used the same ten emotions on the same set of Likert scales as presented to the participants. Accuracy was calculated based on the absolute difference between participants’ ratings and the target’s own ratings, across each of the ten emotion rating scales (larger absolute differences indicate lower accuracy; for a similar approach see Eyal et al. 2018; Zhou et al. 2017). We used the average accuracy score across all four targets as the unit of analysis, consistent with previous research on empathic accuracy and emotion recognition (e.g., Eckland et al. 2018; Israelashvili et al. 2020a; Mackes et al. 2018; Zaki et al. 2008), and consistent with the average measure used in other recognition tasks (RMET, GERT). Finally, to simplify the interpretation of this index, the average absolute difference was reversed (−1* average absolute difference), such that a higher score reflects better accuracy. The average absolute difference between the predicted intensity of emotions of the storytellers and their actual self-ratings across the four videos in Study 1 was 18.3 (SD = 4.4; intraclass correlation = 0.94) and in Study 2 15.82 (SD = 3.22; intraclass correlation = 0.89). Admittedly, the measure of accuracy based on absolute differences scores is not always suitable, particularly when the resulted measure has poor reliability and scores are difficult to interpret (e.g., Peter et al. 1993). However, in the present study, the reliability of the measure was good. We also believe that the difference scores neatly capture the degree of agreement between perceivers’ ratings of targets’ emotions with targets’ self-reported emotion ratings. Nonetheless, we also applied an alternative calculation of accuracy based on the correlation (rather than absolute difference) between the participants’ ratings and the target’s own ratings. The findings of both methods were consistent (see Supplemental Materials). 

Verbal intelligence (Verbal IQ). To assess verbal intelligence, we used the Shipley Vocabulary Test (Shipley 1940). For each item, participants are instructed to decide which of four words is most similar to a prompted word. The original version of the test includes forty items; here we used the twenty first items. Verbal IQ was determined by calculating the percentage of correct responses across all twenty items. The average percentage of correct answers in Study 1 was 67% (SD = 18%; Cronbach’s α = 0.78) and in Study 2 was 78% (SD = 16%; Cronbach’s α = 0.72).

Enjoyment. After each recognition task, participants were asked to rate how much they had enjoyed the task. Answers were provided on a 7-point Likert scale ranging from 0 = *not at all* to 6 = *very much*. A measure of *enjoyment* was calculated separately for each task for use in the analysis reported below. The average enjoyment across all three recognition tasks was relatively high (Study 1: *M* = 4.27, SD = 1.37; Study 2: *M* = 3.98, SD = 1.19).

### 2.3. Procedure 

Participants thus completed three emotion recognition tests: the Reading the Mind in the Eyes Test (RMET), the Geneva Emotion Recognition Test (GERT), and the Emotional Accuracy Test (EAT). In Study 1, a technical problem resulted in the EAT being presented first, followed by the RMET and the GERT in randomized order. In Study 2, all three tests were presented in randomized order. In both studies, all tests were presented without time restrictions. After each recognition test, participants rated how much they enjoyed taking that task before proceeding to the next task. Finally, we administered the Verbal IQ task^5^. 

## 3. Results

**Figure 1:**
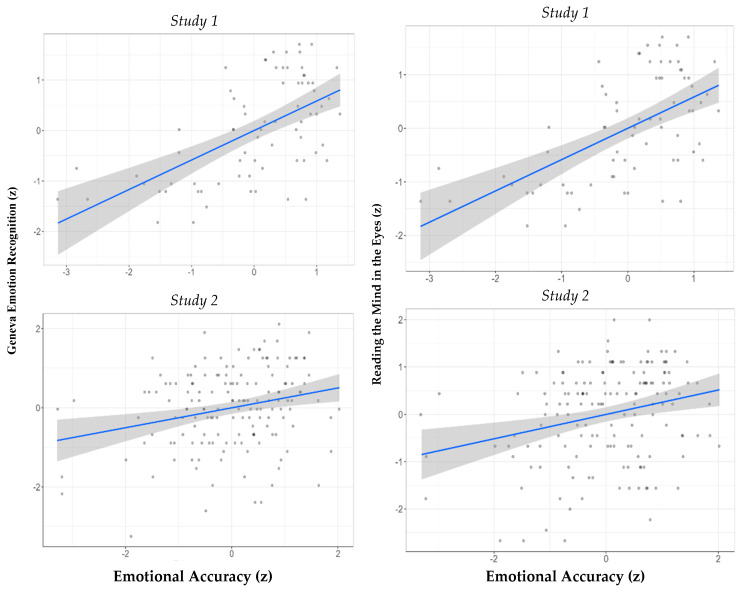
The relationship between accurate emotion recognition on the EAT and the GERT (**left**) and the RMET (**right**), in Study 1 (upper panel) and Study 2 (lower panel). *Note.* Grey denotes 95% confidence intervals.

Emotion recognition performance. Our first hypothesis was that performance on the three recognition tests would be significantly positively correlated. Since the variables in Studies 1 and 2 were not normally distributed (Shapiro–Wilk test > 0.90, *p* < 0.001; see distributions in the Supplemental Materials), we used the Spearman correlation coefficient. However, in keeping with our preregistered analysis plan, we also provided the results for the Pearson correlations. Table 2 presents the bivariate correlations of performance on the three tests. The findings of both studies show that, as predicted, individuals who performed better on the EAT also performed better on the GERT and the RMET (see Figure 1). 

We also found that the performance on all three tests was correlated with verbal IQ (see Table 2). Accordingly, we exploratorily examined correlations between the three recognition tasks while statistically controlling for individual differences in verbal IQ. Findings showed that performance across all three tests remained positively significantly correlated above and beyond their link to verbal IQ (for Study 1: *r* > 0.5, *p* < 0.001; for Study 2: *r* > 0.2, *p* < 0.02, see Supplemental Table S1). 

Enjoyment of taking the test. Our second hypothesis was that participants would report enjoying the EAT more than the other tasks (GERT, RMET). To test this hypothesis, we conducted a one-way analysis of variance (ANOVA) with a repeated measure. We entered the test (EAT, RMET, GERT) as the within-subject factor, and enjoyment as the dependent variable. We utilized Greenhouse–Geisser correction to adjust ANOVA values for sphericity, and we used Bonferroni correction to adjust the significance levels of all follow-up analyses to account for multiple comparisons. 

Both studies found significant differences in enjoyment across tests (see Table 3): Study 1: *F* (1.7, 124.7) = 18.6, *p* < 0.001; η_p_^2^ = 0.205; Study 2: *F* (1.9, 289.7) = 3.67, *p* = 0.03; η_p_^2^ = 0.023. Follow-up analyses indicated that participants enjoyed taking the EAT significantly more than taking the RMET: Study 1: *t*(72) = 3.96, *p* < 0.001, Cohen’s d = 0.46; Study 2: *t*(156) = 2.14, *p* = 0.034, Cohen’s d = 0.17. Findings also showed that participants enjoyed taking the EAT more than taking the GERT in Study 1: *t*(72) = 5.17, *p* < 0.001, Cohen’s d = 0.61, and comparable to the GERT in Study 2: *t*(156) = 0.17, *p* = 0.87, Cohen’s d = 0.01. The findings from Study 1 thus fully, and Study 2 partially, support Hypothesis 2, demonstrating that while participants found all three tests quite pleasant, they tended to enjoy the EAT more than the other tests. 

Comparing the results of Study 1 and 2. Our results point to consistent individual differences in performance across emotion recognition tasks. While the *direction* of the effect was significant and positive across both Studies 1 and 2, the *strength* of the observed effect in Study 2 (*r* = 0.22) was significantly lower than that in Study 1—(*r* = 0.55) Z = 2.75, *p* = 0.01—even though we utilized identical criteria for data-cleaning and analysis. We do not have a theoretical explanation for this difference; we consider it likely that it may reflect variance in the true effect size between different studies (i.e., sampling error). The reliabilities of the measurements obtained in Study 2 also were lower than in Study 1, which can partly account for differences in the correlations (i.e., lower reliability sets a lower boundary for the maximal correlation between two measurements; see Schmitt 1996; Kenny 2013). In addition, there is a general tendency of studies with American participants to show stronger effects than those using samples from other countries (e.g., Fanelli and Ioannidis 2013), pointing to a potential cultural difference. 

The estimations of correlations observed in Study 2 were more in the range of the average correlations observed in previous studies reviewed by Schlegel et al. (2017; *r* = 0.29 for the relation between performance on different nonverbal recognition tests and *r* = 0.15 for the relation between posed and spontaneous recognition tests). Thus, while Studies 1 and 2 showed different strengths of effects, both clearly point to significant positive correlations between performance on different recognition tests, which aligns with the results from the meta-analysis of Schlegel and colleagues (2017). 

## 4. Discussion

Across two independent samples, we investigated the convergent validity of a newly developed emotion recognition measure, the Emotional Accuracy Test (EAT). This test uses spontaneous rather than prototypical, posed expressions as stimuli and examines emotion recognition in terms of the agreement between perceivers’ ratings of targets’ emotions with targets’ self-reported emotion ratings. Convergent validity of the EAT was assessed by comparing performance on the EAT with scores on two well-established measures of emotion recognition ability that employ static (RMET) and dynamic (GERT) posed or enacted nonverbal expressions. We found support for our preregistered hypothesis, demonstrating that individuals’ performance broadly aligns across these three different tasks. This finding remained robust even when individual differences in verbal IQ were statistically controlled, suggesting that the interrelations between these different recognition tests were not merely due to individual differences in verbal IQ. Furthermore, we found that participants reported significantly more enjoyment of the EAT compared with the RMET (Studies 1 and 2) and the GERT (Study 1). 

The current research complements and extends existing knowledge by showing that individuals’ ability to recognize others’ emotions is relatively consistent, not only among nonverbal tasks but also when comparing performance across dramatically different emotion stimuli. The stimuli involved in each test differed on several important features: containing only non-verbal information vs. verbal and nonverbal information, posed or enacted vs. spontaneous expressions, and brief displays vs. several minutes. Moreover, accuracy was defined on the basis of different criteria across the tasks. In the EAT, the criterion for accuracy is agreement with the subjective self-report of emotional experience by the person who shared the story, while in the other two tests, the criterion for accuracy is agreement with the researchers (RMET) or the intended emotion (GERT). Nevertheless, on average, individuals who performed better on one task also performed better on the other tasks. On a practical level, our finding suggests that performance, as assessed using established paradigms frequently used to measure the recognition of non-verbal emotional expressions, do constitute valid proxies to understanding others’ emotions in more realistic settings. This conclusion, however, only partly aligns with existing research. For example, one previous study found that performance on emotion recognition tests using prototypical expressions was not correlated with accuracy in recognizing the valence of spontaneous emotional expressions portrayed during naturalistic dyadic interactions (Sze et al. 2012). The lack of association could be attributed to differences between recognizing multiple discrete emotions with varying intensities (as in the current paper) vs. recognizing valence (as in Sze et al. 2012; but see also Brown et al. 2018). Thus, future research is needed to clarify under what conditions non-verbal and prototypical emotional expressions constitute valid proxies to understanding others’ emotions complex real-world settings.

Other factors might also contribute to the positive relation between the three tests, including the activation of shared cognitive processes and the reliance on language. Connolly and colleagues (2020) noted that understanding the meaning of emotional expressions makes demands on working memory. It requires holding all expressive cues in mind while attending to response options in order to make a judgment. When participants are unsure about the correct response, they may be able to use cognitive strategies (e.g., method of elimination) to decipher the intended expression. Given that all three tests require participants to make judgments of emotion stimuli, shared cognitive resources may explain part of the associations we found. Another possible explanation for the correlation across recognition tests might be that all tasks require an understanding of language to complete the test. The relation between the three tests may thus result from their association with verbal IQ (see Jones et al. 2011; Tang et al. 2020). To directly account for this possibility, we exploratorily examined correlations between the three recognition tasks when controlling for individual differences in verbal IQ. Findings showed that performance across all three tests remained positively and significantly correlated. This finding provides evidence that the ability to understand others’ emotions accurately is related to, yet separate from, vocabulary per se. 

Finally, previous research has shown that spontaneous expressions are more recognizable when they are more prototypical (Sauter and Fischer 2018). The stimuli used in this paper reflected how people share their emotional experiences in daily life, and thus the present stimuli are really different in nature from the posed, short and prototypical stimuli that are used in most other tests. Still, it is possible that the spontaneous expressions displayed in the videos included momentary prototypical emotions that our participants could have used to accurately rate the targets’ emotions, which might also contribute to the positive relation between the three tests.

Intriguingly, we also found differences in the strength of associations (across the three tests) between the US vs. UK sample. Future research will be needed to examine whether such differences are consistent and how they can be explained. For example, cultural distinctions might reflect differential familiarity with the tests, or indirectly result from cultural differences in response biases for rating scales (e.g., Lee et al. 2002).

We found that participants enjoyed taking the EAT more than other tasks in Study 1. However, this large effect might have reflected the fact that participants completed the EAT before the other two tests. In Study 2, all tasks were presented in random order, and we observed a small, yet significant effect showing that participants enjoyed the EAT and the GERT (both tests with dynamic stimuli and spontaneous or enacted expressions) more than the RMET (which uses static stimuli, posed expressions). The findings of both studies thus suggest that participants’ enjoyment of the EAT is equal to or higher than the other two tests. Each stimulus of the EAT consists of a person sharing a real emotional experience from their own life, arguably making these stimuli highly relatable. The content of the emotional experiences and the individuals sharing their experiences were different for each stimulus, ensuring variability for participants. Although repetitive elements of test environments are designed to reduce cognitive demand, confusion, and distractions, they might also reduce enjoyment. We posit that the enjoyment participants experience when completing the task may help some individuals to concentrate more and perform better. 

The present study was not intended to test whether the EAT is a *better* test than other emotion recognition tests. Different approaches have different pros and cons. Using more standardized, posed repetitive stimuli allows researchers to select a single communication channel (e.g., eyes) and to control many features of the stimuli. However, low ecological validity may be a concern for studies with more relational aims (e.g., the role of shared life experiences for understanding others’ emotions; see Israelashvili et al. 2020a). The choice of test must depend on the research question at hand; the EAT offers an additional emotion recognition tool with a unique set of features that we hope will be useful to researchers interested in emotion recognition. 

Nevertheless, we also want to acknowledge some limitations of the EAT. Firstly, the videos feature negative emotional events shared by female targets. We used female targets because previous research has found that women tend to share their feelings more extensively than men (e.g., Rimé et al. 1991), and to minimize individual differences unrelated to the main research question. Further research is needed to test whether the positive relation between performance on the EAT and the GERT and RMET would be observed with other targets (e.g., men) and with different emotional content (e.g., positive stories) and languages other than English. As the GERT and the RMET are not limited to negative emotions nor female targets or any verbal content, we expect the pattern of results to hold. We thus speculate that the shared underlying ability to understand others’ emotions is activated across different emotions (positive and negative) and different targets (men and women). Yet, a robustness check for the conclusion that emotion recognition tests using prototypical expressions are valid proxies for spontaneous expressions would be best achieved by replicating the present findings using targets with diverse levels of expressivity and variability in emotion intensity.

Another limitation is that we operationalized accuracy of emotion recognition as a match between participants’ and targets’ ratings. Naturally, targets themselves may not have been accurate in assessing their own emotions; thus, accuracy may be less objective than the term suggests. However, in the realm of interpersonal understanding, the target’s reports of how they felt may be more relevant than some objective established criteria when operationalizing emotion recognition accuracy. 

How might the targets have generated the ratings of their own emotions, subsequently used as the “ground truth” in the calculation of perceiver–target agreement of the EAT. Targets were instructed to provide ratings of the emotions they expressed in the video, rather than in the situation described in the story. The emotional judgments were likely also based on the recollection of the emotions the targets experienced when the original event happened. We presume that targets’ self-rating indeed represents how they experienced their emotional state at the time of the video, and therefore, that the Emotional Accuracy Test measures the agreement between emotions experienced by the self vs. those perceived by a third party.

In sum, we believe that the EAT has both ecological validity (real emotional stories) and convergent validity (convergent pattern with external measures of performance), making it appropriate for measuring the understanding of others’ emotions. Future studies will be needed to establish additional psychometric properties of the EAT, including test–retest reliability and discriminant validity. 

## 5. Conclusions

A frequently raised concern with emotion recognition tests that use posed prototypical emotion expressions is their ecological validity, and thus whether they are useful in predicting emotion understanding in daily life settings. We, therefore, developed a new emotion recognition test, the Emotional Accuracy Test (EAT), using more spontaneous and natural emotional stimuli. Our findings show that the EAT is positively correlated with two other emotion recognition tests using prototypical expressions but is more pleasant for participants. Thus, we suggest that researchers have considerable degrees of freedom in choosing which test to use, depending on the goal of their research.

## Figures and Tables

**Table 1:** Description of emotion recognition tasks.

Task	Stimuli	Emotional Cues	Emotional Expression	Basis of Accuracy^3^	Choice Options
RMET	Static pictures	Eyes (nonverbal)	Posed	Prototypical expression	Four (select one)
GERT	Dynamic videos	Voice, body and face (nonverbal)	Reenacted	Prototypical expression	Fourteen (select one)
EAT	Dynamic videos	Words, voice, facial and body movements (verbal and nonverbal)	Spontaneous	Targets’ emotions	Ten (select all applicable, rate each using 0–6 scale)

*Note.* EAT, Emotional Accuracy Test; GERT, Geneva Emotion Recognition Test; RMET, Reading the Mind in the Eyes Test. An additional feature relevant to the stimuli is that the pictures of the RMET are all black and white, while the videos in the GERT and the EAT are all colorful. An additional feature relevant to the choice options is that in the RMET, every stimulus face is paired with a different four choice options, while in the GERT and the EAT, all stimuli use the same fourteen (GERT) or ten (EAT) choice options.

**Table 2:** Pearson and Spearman rho correlation coefficients for the associations of performance as measured across pairs of tasks, in Study 1 and 2.

***Study 1* (N = 74; USA, MTurk)**							
***Pearson’s r***	**EAT**	**GERT**	**RMET**	***Spearman’s rho***	**EAT**	**GERT**	**RMET**
GERT	0.59 *** (0.41, 0.72)			GERT	0.55 *** (0.37, 0.69)		
RMET	0.60 *** (0.43, 0.73)	0.65 *** (0.49, 0.76)		RMET	0.55 *** (0.37, 0.69)	0.65 *** (0.49, 0.77)	
Verbal IQ	0.31 *** (0.09, 0.51)	0.37 *** (0.15, 0.55)	0.45 *** (0.24, 0.61)	Verbal IQ	0.39 *** (0.18, 0.57)	0.34 *** (0.12, 0.53)	0.45 *** (0.25, 0.62)
**Study 2 (N = 157; UK; Prolific)**							
***Pearson’s r***	**EAT**	**GERT**	**RMET**	***Spearman’s rho***	**EAT**	**GERT**	**RMET**
GERT	0.25 ** (0.10, 0.39)			GERT	0.22 ** (0.07, 0.36)		
RMET	0.26 ** (0.11, 0.40)	0.34 *** (0.19, 0.47)		RMET	0.25 ** (0.10, 0.39)	0.25 ** (0.10, 0.39)	
Verbal IQ	0.15 (−0.01, 0.30)	0.33 *** (0.18, 0.46)	0.29 *** (0.14, 0.43)	Verbal IQ	0.04 (−0.12, 0.20)	0.24 ** (0.09, 0.38)	0.25 ** (0.10, 0.39)

*Note.* All patterns of significant positive correlations between the three tasks remained the same when variance explained by Verbal IQ was partialled out (see Table S1 in the Supplemental Materials). EAT—Emotional Accuracy; GERT—Geneva Emotion Recognition Test; RMET—Reading the Mind in the Eyes Test; Verbal IQ; * *p* < 0.05; ** *p* < 0.01; *** *p* < 0.001; 95% confidence intervals (lower, upper).

**Table 3:** Mean (and SD) values of enjoyment participants reported for the completion of the EAT vs. the RMET vs. GERT tasks, in Study 1 (USA) and Study 2 (UK).

	EAT	GERT	RMET
*Study 1*	4.77 ^a^ (1.21)	3.85 ^b^ (1.85)	4.16 ^b^ (1.59)
*Study 2*	4.09 ^a^ (1.41)	4.07 ^a^ (1.53)	3.78 ^b^ (1.56)

*Note.* EAT—Emotional Accuracy Test; GERT—Geneva Emotion Recognition Test; RMET—Reading the Mind in the Eyes. Within each study, numbers that do not share a superscript differ significantly at *p* < 0.05, with Bonferroni correction.

## Data Availability

The data presented in this study are available on request from the corresponding author.

## Notes

1 Noteworthy is the classical, dyadic version of the empathic accuracy paradigm (e.g., Ickes et al. 1990; Stinson and Ickes 1992). One limitation of this original version of the paradigm is that the paradigm was used each time with new target individuals. There was thus no standard test to utilize across studies. Here, we focus our discussion on a more recent version of the empathic accuracy paradigm, which involves a standard set of target individuals to be utilized across different studies, making each finding directly comparable to previous findings using the same stimulus set.

2 The *Emotional Accuracy Test* discussed in the present manuscript is identical to the recognition test originally described in Israelashvili et al. (2020a): which referred to *accurate emotion recognition*.

3 Readers should note that some emotion categories in these tasks (GERT, RMET) do not have a prototypical expression (e.g., playful). Nonetheless, we refer to them as prototypical since we presume that resemblance with prototypical (rather than idiosyncratic) representations of emotional expressions guided the production (GERT) and the selection (RMET) of all emotional stimuli included in these tests.

4 This inclusion criterion was not preregistered for Study 1. Our decision to nevertheless apply it was primarily informed by reviewers’ comments about the need to report only reliable data. Importantly, all patterns of findings reported in this manuscript remain the same (or stronger) when the excluded participants are included in the analyses.

5 Participants also completed the Interpersonal Reactivity Index (IRI; Davis 1983) and the Ten Items Personality Inventory. We also asked whether participants had had similar life experiences to those described in the videos and assessed their empathic responses toward the person in the video by eliciting written support messages. These measures were collected for research questions not addressed in the present manuscript. Here, we focus on measures and analyses directly relevant for testing our hypotheses, as specified in the preregistration of the current study.

## Supplementary Materials

The following are available online at https://www.mdpi.com/article/10.3390/jintelligence9020025/s1, Figure S1: Illustration of emotional accuracy based on the absolute difference between attributed emotions and emotions of the target, Figure S2: Key variables, their distributions, and interrelations for Studies 1 and 2, Table S1: Interrelations of key variables when controlling for verbal IQ scores.
