# Supplementary material for: Emotion Recognition from Realistic Dynamic Emotional Expressions Cohere with Established Emotion Recognition Tests: A Proof-of-Concept Validation of the Emotional Accuracy Test
Source: J Intell. 2021 May 7;9(2):25. doi: 10.3390/jintelligence9020025 (PMC8162550; doi:10.3390/jintelligence9020025)
Supplement: Supplementary file 1 [file jintelligence-09-00025-s001.zip › jintelligence-1047516-supplementary materials.pdf]

Supplemental materials

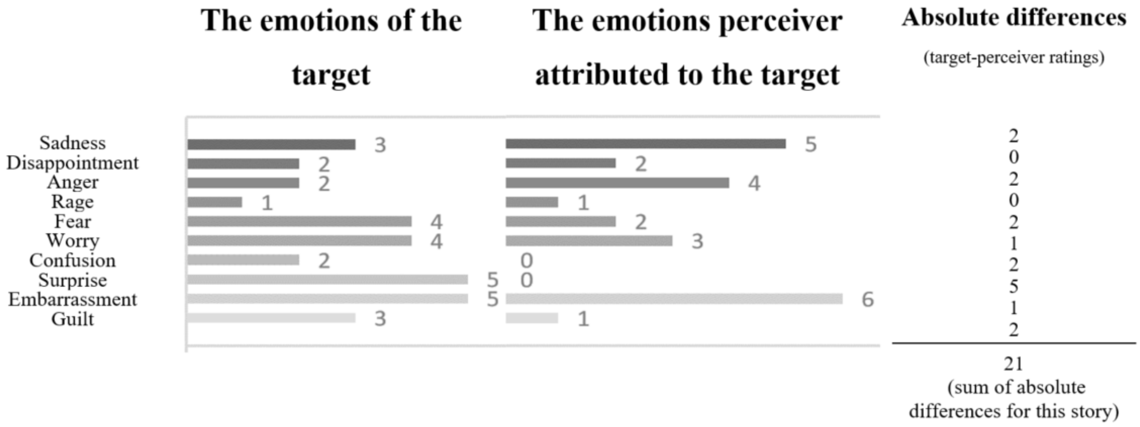

Figure S1. Illustration of emotional accuracy based on the absolute difference between attributed emotions and emotions of the target (larger absolute differences indicate smaller accuracy).

Findings using an alternative measure of accuracy

In Studies 1 & 2 we calculated the performance on EAT based on the absolute difference, between a target's reported emotions and a perceiver's inference of these emotions. Yet, an alternative calculation can be done based on the correlation between a target's reported emotions and a perceiver's inference of these emotions. We conducted this calculation of the correlation and found that the measure of accuracy based on *correlation* is consistently and significantly positively associated with accuracy as calculated, using the *absolute difference* score (Study 1: $r = 0.84^{***}$, Study 2: $r = 0.67^{***}$). We then used the (Fisher transformed) correlation measure to re-analyze our main findings. This exploratory analysis showed that performances on the three recognition tests were significantly positively correlated (Study 1: $r > .61^{***}$; Study 2: $r > .24^{**}$; see below details of all specific correlations). Thus, the hypothesized positive relations between different measures of emotion recognition is supported under the preregistered definition of accuracy as absolute difference, and even under the exploratory definition of accuracy as correlation. To be consistent with our preregistration, in the main text we report the results based on the original *absolute difference* score.

Controlling for Verbal IQ

Pearson correlation coefficients for the associations between performance as measured across pairs of tasks with and without controlling for Verbal IQ. Findings show that the patterns of significant associations all remained robust.

Study 1

		EAT	GERT	RMET	Verbal IQ
Without control	EAT	1	.59***	.60***	.31***
			[.42, .72]	[.43, .73]	[.09, .50]
	GERT		1	.65***	.37***
				[.49, .77]	[.15, .55]
	RMET			1	.45***
					[.25, .62]
	Verbal I.Q.				1
With controlling for Verbal IQ	EAT	1	.53***	.54***	
			[.34, .68]	[.35, .68]	
	GERT		1	.58***	
				[.40, .71]	
	RMET			1	

Study 2

		EAT	GERT	RMET	Verbal IQ
Without control	EAT	1	.25**	.26**	.15
			[.10, .39]	[.11, .40]	[-.01, .30]
	GERT		1	.34***	.33***
				[.19, .47]	[.18, .46]
	RMET			1	.29***
					[.14, .43]
	Verbal I.Q.				1
With controlling for Verbal IQ	EAT	1	.22**	.23**	
			[.07, .36]	[.08, .37]	
	GERT		1	.28***	
				[.13, .42]	
	RMET			1	

Table S1.

Note. *** $p < .001$; EAT – Emotional Accuracy Test; GERT – Geneva Emotion Recognition Test; RMET – Reading the Mind in the Eyes Test; Verbal IQ; 95% Confidence Intervals [lower, upper].

Key variables, their distributions, and interrelations for Study 1 & 2

Study 1

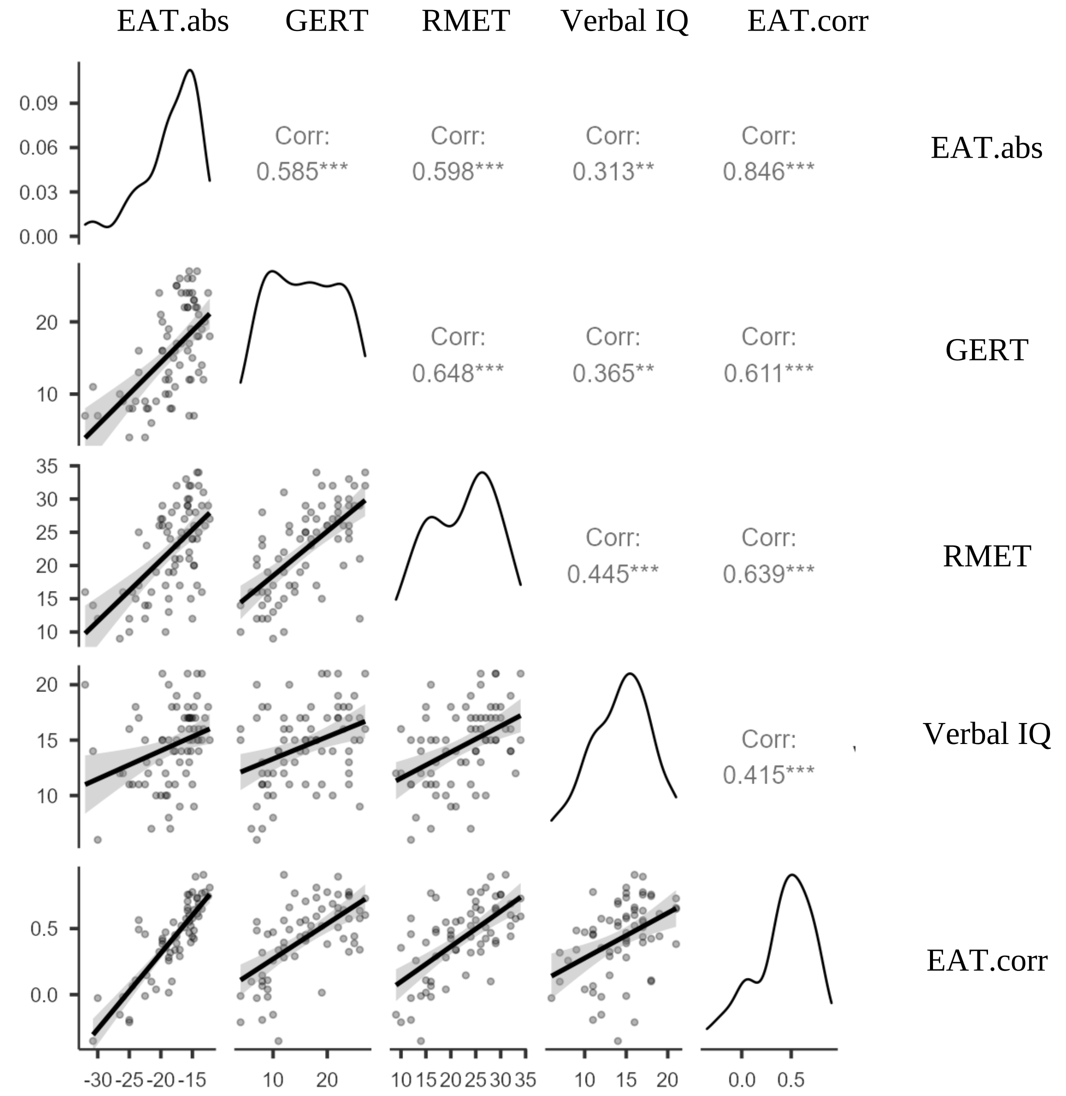

Figure S2.1 Key variables, their distributions, and interrelations for Study 1.

Note. EAT.abs – Emotional Accuracy Test (based on absolute difference score); EAT.corr – Emotional Accuracy Test (based on correlation score); GERT – Geneva Emotion Recognition Test; RMET – Reading the Mind in the Eyes Test; Verbal IQ.

Study 2

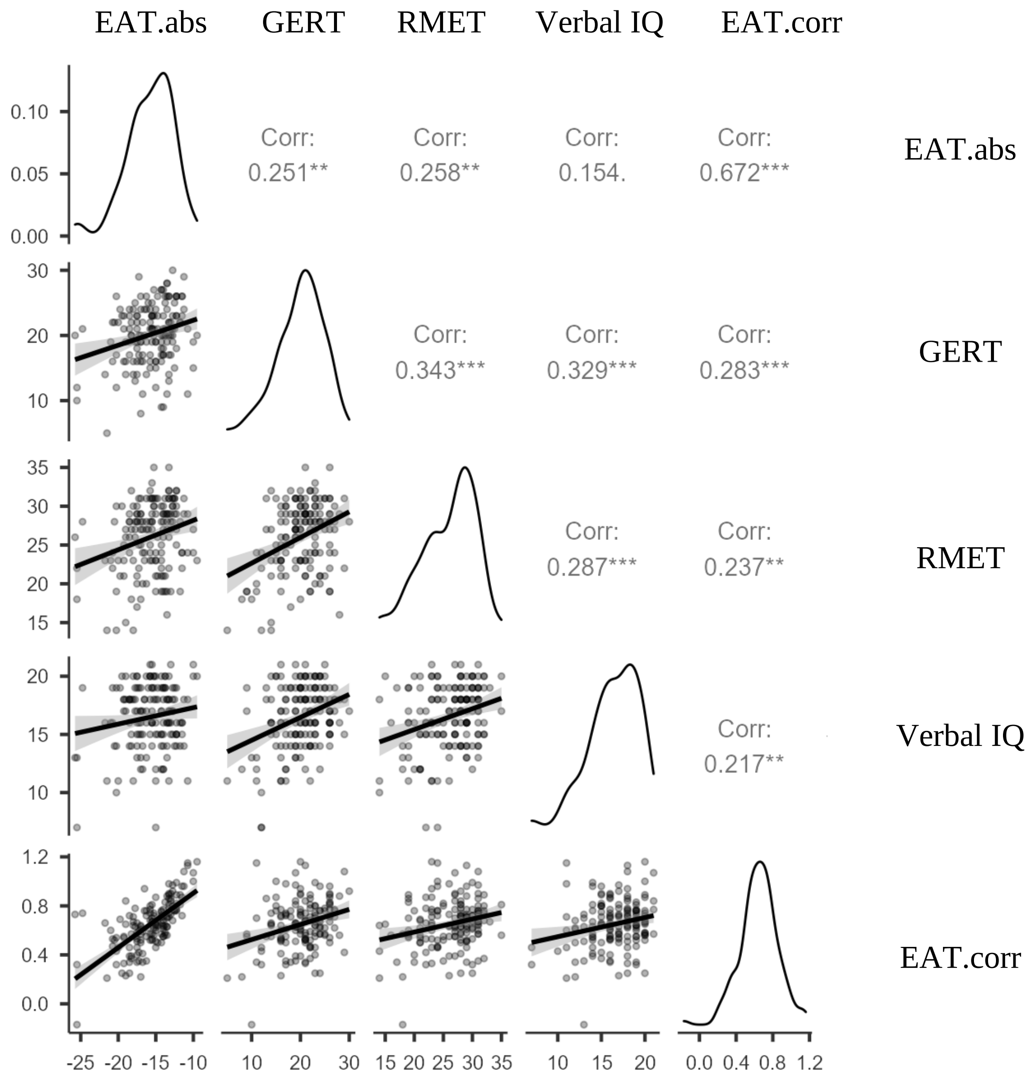

Figure S2.2 Key variables, their distributions, and interrelations for Study 2.

Note. EAT.abs – Emotional Accuracy Test (based on absolute difference score); EAT.corr – Emotional Accuracy Test (based on correlation score); GERT – Geneva Emotion Recognition Test; RMET – Reading the Mind in the Eyes Test; Verbal IQ.

Associations of the EAT with the IRI questionnaire

At the end of the research, we also collected data on a self-reported measure of dispositional empathy (Davis, 1983), involving the subscales of Empathic Concern, Personal Distress, Fantasy, and Perspective Taking. Pearson correlation coefficients for the associations between performance on the EAT and these four subscales were in Study 1 r = .06, -.37***, .00, .01; respectively), and in Study 2 r = .08, -.07, .03, .09; respectively).
